# Self-Assembling Complexes of Quantum Dots and scFv Antibodies for Cancer Cell Targeting and Imaging

**DOI:** 10.1371/journal.pone.0048248

**Published:** 2012-10-25

**Authors:** Tatiana A. Zdobnova, Oleg A. Stremovskiy, Ekaterina N. Lebedenko, Sergey M. Deyev

**Affiliations:** 1 Institute of Bioorganic Chemistry of the Russian Academy of Sciences, Moscow, Russia; 2 Department of Biology, Lobachevsky State University Nizhny Novgorod, Nizhny Novgorod, Russia; 3 Research Institute of Applied and Fundamental Medicine, Nizhny Novgorod State Medical Academy, Nizhny Novgorod, Russia; Cincinnati Children's Hospital Medical Center, United States of America

## Abstract

Semiconductor quantum dots represent a novel class of fluorophores with unique physical and chemical properties which could enable a remarkable broadening of the current applications of fluorescent imaging and optical diagnostics. Complexes of quantum dots and antibodies are promising visualising agents for fluorescent detection of selective biomarkers overexpressed in tumor tissues. Here we describe the construction of self-assembling fluorescent complexes of quantum dots and anti-HER1 or anti-HER2/neu scFv antibodies and their interactions with cultured tumor cells. A binding strategy based on a very specific non-covalent interaction between two proteins, barnase and barstar, was used to connect quantum dots and the targeting antibodies. Such a strategy allows combining the targeting and visualization functions simply by varying the corresponding modules of the fluorescent complex.

## Introduction

Among the major methods of fluorescent visualization of tumors is the one based on detection of selective biomarkers overexpressed in tumor tissues that allows revealing the tumor type, metastatic processes, tumor drug resistance, etc. [1]. Contrasting agents used for this purpose generally consist of two parts, or modules: a visualizing module that is responsible for target detection and a targeting one that selectively binds to a certain cell type.

In the past decade, fluorescent semiconductor nanocrystals, referred to as quantum dots (QD), have attracted much attention as visualizing agents for biological applications. Among the most advantageous properties of QD are the remarkable brightness of fluorescence, photostability, wide excitation and narrow emission spectra, and a rich palette of spectrally tunable emission bands, etc. These properties enable multicolor labeling and the simultaneous identification of various biological objects as well as long-term bio-imaging [2].

As a targeting module, scFv antibodies appeared to be more promising for both *in vitro* and *in vivo* applications [3]. The scFv antibodies consist of a single polypeptide chain combining variable domains of immunoglobulin light and heavy chains that are connected via a peptide linker. Such antibody derivatives can be produced in bacterial expression systems as stable proteins retaining antigen specificity of a full-length antibody, yet lacking the Fc domain that is responsible for the effector function of immunoglobulins and is generally undesirable for in vivo targeting applications.

In this work, for model antibodies (as a targeting module), we chose anti-tumor 425scFv [4] and 4D5scFv [5], which selectively bind to oncomarkers HER1/EGFR and HER2/neu, respectively. These oncomarkers are trans-membrane proteins from the family of the epidermal growth factor receptors that are overexpressed in many tumor cells and have a great diagnostic and prognostic significance [6]. Previously, these scFvs have been successfully used for targeted delivery of fluorescent proteins and therapeutic agents to tumor cells [7], [8], [9], [10].

At present, there are two approaches of QD conjugation with targeting agents: direct conjugation and conjugation via adaptor molecules. Direct conjugation is not an optimal method because targeting agents are altered during the conjugation procedure. For example, antibodies conjugated to QD retain their antigen specificity but their affinity may significantly decrease. [11]. Furthermore, direct conjugation of QD to a targeting antibody requires testing the activity of the antibody in each particular case.

The use of self-assembling adaptors – small and ‘sticky’ molecules, effectively and specifically binding to each other without formation of homodimers, appears to be a more promising method of binding the targeting antibody to QDs. In this work, we present the barnase-barstar system (BBS) as a universal tool for producing fluorescent complexes of different selectivity and parameters of fluorescence on the basis of QDs and scFv antibodies for visualization of tumor cells.

## Materials and Methods

### Bacterial expression and purification of recombinant proteins

The mutant barstar C40/82A (herein referred to as barstar), wild-type barnase [7], [12], recombinant anti-HER2/neu 4D5scFv and anti-HER1 425scFv antibodies [13] as well as (4D5scFv)_2_-Bn fusion protein [14] were produced in *Escherichia coli* and purified as described previously [7].

**Figure 1:**
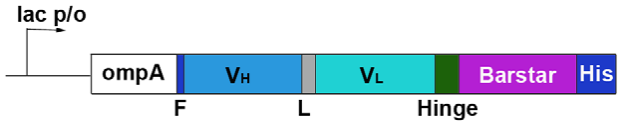
Gene construct encoding the 425scFv-Bs recombinant protein. The 425scFv-Bs-His_6_ construct starts with an N-terminal short FLAG tag (F, dark blue) followed by 425scFv in V_H_-linker-V_L_ orientation (V_H_, cyan; L, gray; V_L_, turquoise), 16-amino-acid hinge linker (green), barstar (purple). The construct terminates with a His_6_-tag (dark blue) attached to the C-terminus of 425scFv-barstar fusion protein. The fusion gene is under control of the *lac* promoter. *OmpA* – the signal peptide for directed secretion of the recombinant protein to the *E. coli* periplasm.

The expression plasmid for 425scFv-Bs fusion protein was constructed on the basis of the pSD-4D5scFv-barstar plasmid [15] (*figure 1*). Genetic engineering manipulations, cell culturing and cell lysis were performed according to standard protocols. The DNA fragment encoding 425scFv protein was amplified from a pKM30425M1ChCl plasmid [4] using primers 5′GACTCGATATCGAAGTGCAACTGCAGCAGTC and 5′CTGTGGAATTCCCGTTTGATCTCCAGTTCTG. The product of amplification was cloned into pSD-4D5scFv-barstar plasmid instead of 4D5scFv gene using EcoRV and EcoRI restriction endonucleases. The resulting pSD-425-Bs-His_6_ construct was verified by sequencing.

For production of 425scFv-Bs containing His_6_-tag on *C*-terminus, the *Escherichia coli* strain BL21 was transformed with pSD-425-Bs-His_6_ and grown in lysogeny broth (LB) at 28°C. The 425scFv-Bs expression was induced by addition of 0.5 mM IPTG at an OD_550_ of 0.8. The bacteria were then incubated at 28°C for 12 h. The cells were harvested, centrifuged, and the pellet was re-suspended in lysis buffer (0.01 M Tris-HCl, pH 8.3, with 0.1 M NaCl and 10 mM EDTA) and sonicated on ice. The lysate was then centrifuged at 22,000 g for 30 min at 4°C. The pellet was used for purification of His_6_-tagged protein on Ni^2+^-NTA column (Qiagen) under denaturing conditions according to the manufacturer's instructions. The protein was denatured with 8 M urea, refolded for 5 h using a linear gradient from 8 to 0 M urea and eluted with 250 mM imidazole. For final purification of 425scFv-Bs, elution fractions were diluted 20-fold, applied onto Q Sepharose FF 1-ml column (GE Healthcare) and eluted using linear gradient from 0 to 500 mM NaCl.

SDS/PAGE analysis of the proteins was performed according to standard protocols using 14% (for barnase and barstar) or 12.5% (for the other recombinant proteins) polyacrylamide gels.

### QD conjugation

QDs with fluorescence emission maximum at 565 or 605 nm were used for the design of the visualizing module. Carboxyl group-coated QDs (Qdot 565 ITK™ carboxyl quantum dots and Qdot 565 ITK™ carboxyl quantum dots, Invitrogen) were conjugated with either of the BBS proteins using EDC/NHS coupling chemistry. 2 µM QDs in 0.1 M MES (pH 6.0) and 0.5 M NaCl were first activated with EDC (∼2 µM) and NHS (∼5 µM) at room temperature for 15 min. The reaction mixture was applied onto Sephadex G-25 column and eluted with a buffer containing 40 mM Na2B4O7 and 30 mM KH2PO4, pH 8.0. Next, eluted activated particles were mixed with barstar or barnase dissolved in the same buffer and incubated for 1 h at room temperature. The reaction mixture was applied onto Sephadex G-25 column and unbound proteins were eluted with PBS (pH 7.4). The QD:protein molar ratios were optimized for each protein.

### Agarose gel electrophoresis

Electrophoresis of QDs and QD-conjugates was performed using 1% agarose gel in TAE buffer (50 mM tris-acetate, 1 mM EDTA, pH 7.6) at 10 V/cm for 30 min. QDs were diluted in TAE and mixed with 6× loading buffer (50% glycerol, 0.1% Bromophenol Blue) before loading onto the gel. Gels were visualized with the Transilluminator Multi Doc-It Digital Imaging system.

### Barstar and barnase activity assay

The ribonuclease activity of barnase, (4D5scFv)_2_-Bn and QD-Bn conjugates was tested with the acid-insoluble RNA precipitation assay described in [16], with the modification of all the solution volumes scaled down 5-fold. The activity of barstar, 425scFv-Bs, and QD-Bs conjugates was assessed by their ability to inhibit the ribonuclease activity of barnase. Barnase at a constant concentration of 26 nM was incubated with serial twofold dilutions of barstar, 425scFv-Bs, or QD-Bs conjugates. The obtained solutions were then used in the Rushizky assay [16].

### Assessment of antibody affinity

Measurements of a dissociation constant were performed using BIAcore instruments (BIAcore 3000). Recombinant p185^HER2-ECD^ or extracellular domain of EGFR (Sino Biological, Inc.) was coupled onto a CM5 chip at density of 4500 RU by standard amine coupling chemistry. All proteins were used at four concentrations (1 µM, 330 nM, 110 nM and 37 nM) in HBS-PE (0.1 M HEPES, pH 7.4, 0.15 M NaCl, 3 mM EDTA, 0.005% Tween-20). The sensograms were obtained at a flow rate of 5 µl/min at 25°C. The dissociation phase lasted for 20 min.

### Cell cultures

The human ovarian adenocarcinoma SKOV-3 (HTB-77, ATCC), human epidermoid carcinoma A431 (CLR-1555), and Chinese hamster ovary CHO (Russian Cell Culture Collection) cells were cultured in McCoy's 5A medium (for SKOV-3) or RPMI-1640 (for A431 and CHO) with 10% (v/v) fetal calf serum (HyClone) and 2 mM *L*-glutamine. Cells were grown in 5% CO_2_ at 37°C.

### Cell labeling and imaging

For HER2/neu-directed cell imaging, SKOV-3 cells overexpressing HER2/neu were plated in 96-wells plates at a density of 2×10^4^ cells per well and cultured overnight. The cells were washed twice with PBS (pH 7.4) before staining. All proteins and QD conjugates were dissolved in PBS (pH 7.4). After a brief washing with cold PBS, the cells were subsequently incubated with the (4D5scFv)_2_-Bn targeting protein at a final concentration of 30 nM and then with 80 nM QD_605_-Bs or QD_565_-Bs conjugates for 40 min at 4°C. After each incubation step, cells were washed three times with cold PBS (pH 7.4). The results of cell labeling were analyzed with an inverted fluorescent microscope Axiovert 200 (Zeiss, Germany). Images were obtained using a CCD camera (AxioCamHRc, Zeiss, Germany) and AxioVision software (Zeiss, Germany).

HER1-directed cell imaging was performed similarly, using HER1-overexpressing A431 cells, 425scFv-Bs targeting module and QD_605_-Bn or QD_565_-Bn visualizing modules.

### Flow cytometry analysis

For flow cytometry analysis, cells were plated in 6-wells plates (Corning) at a density of 4×10^3^ cells per well and cultured overnight. Cells were stained as described above. Stained cells were detached with PBS (pH 7.4) containing 5 mM EDTA and centrifuged. The pellet was re-suspended in 1 ml PBS (pH 7.4) containing 0.1% sodium azide. Cell-associated fluorescence intensity was measured using a FACS Calibur flow cytometer (BD Bioscience) at an excitation wavelength of 488 nm (argon laser). Fluorescence of at least 10,000 cells per sample was analyzed. Cell autofluorescence was estimated using PBS-treated cells as controls.

### *In vitro* cytotoxicity analysis

The cytotoxicity of used QDs, their conjugates and complexes on cell line was assessed using microtitration assay. The SKOV-3 cells were seeded at a density of 4×10^3^ cells per well in a 96-well plate, and were allowed to attach overnight. Then the cells were incubated with PBS (pH 7.4) containing different concentrations of QD probes at 4°C for 1 h. After incubation the cells were washed three times with cold PBS (pH 7.4) and cultured for 48 h in standart conditions. After 48 h, cell viability was estimated by standart MTT assay as described in [9]. The cell viability was expressed as percentage of the optical density of untreated cells from two experiments carried out in triplicate.

## Results

### General strategy: the barnase-barstar system

**Figure 2:**
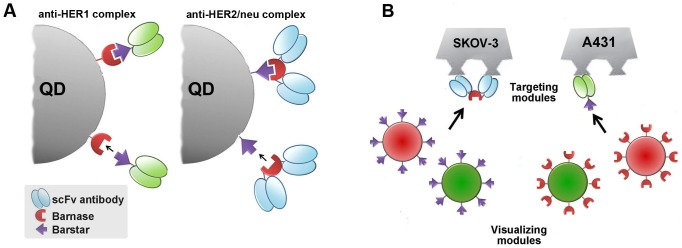
The design of fluorescent probes on the basis of QDs and 425scFv (a, green antibody) or 4D5scFv (b, blue antibody) for specific cancer cell imaging. Binding of QDs to scFv antibodies via barnase-barstar molecular adaptors (**A**) and BBS-based molecular constructor comprising of a set of variable fluorescing and targeting modules (**B**) are shown.

In our previous work, the barnase-barstar system (BBS) was initially developed for antibody multimerization [15], [17]. The bacterial ribonuclease barnase from *Bacillus amyloliquefaciens* and its natural inhibitor barstar are small proteins (12 and 10 kDa, respectively) with extremely high affinity of binding (K_d_∼10^−14^ M) [18], which is comparable with affinity of streptavidin-biotin interaction [19]. Here we utilized these proteins as molecular adaptors to obtain a series of self-assembling fluorescent complexes based on quantum dots with different anti-tumor specificity and color (*Table 1*). The probes are composed of a visualizing module, namely, QDs conjugated to one of the BBS proteins (either barstar or barnase), and a targeting module, i.e., anti-tumor antibodies fused to the partner BBS protein (either barnase or barstar, respectively) (*figure 2A*). The resulting visualizing and targeting modules can be combined in different ways depending on the molecular specificity and optical properties required for a particular bioimaging task (*figure 2B*).

**Table 1:** Self-assembling QD-scFv complexes and their anti-tumor specifities.

No	Complex composition		Complex specificity	Cell staining		
	Targeting module	Visualizing module		SKOV-3 (HER2/neu-overexpressed)	A431 (HER1-overexpressed)	CHO(control)
1	(4D5scFv)2-Bn	QD_605_-Bs	HER2/neu	+	-	-
2		QD_565_-Bs	HER2/neu	+	-	-
4	425scFv-Bs	QD_605_-Bn	HER1	-	+	-
5		QD_565_-Bn	HER1	-	+	-

### Targeting module: construction and characterization of recognition proteins

Anti-HER1 425scFv and anti-HER2 4D5scFv antibodies were used as targeting molecules for directed delivery of QDs to tumor cells. We obtained a number of 425scFv and 4D5scFv fusion proteins with barnase or barstar in different combinations. Two fusion proteins with the highest expression yields were chosen as the targeting modules for QDs delivery: monovalent 425scFv fused to barstar (425scFv-Bs) and divalent 4D5scFv fused to barnase ((4D5scFv)_2_-Bn).

**Figure 3:**
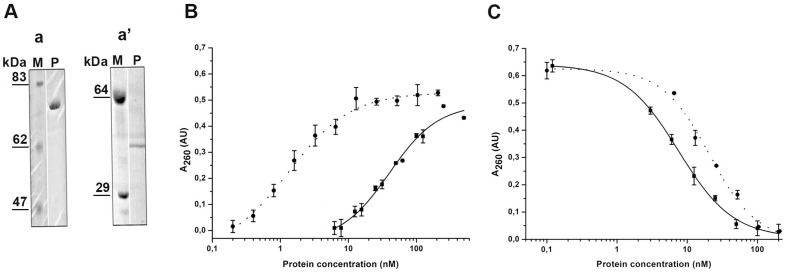
Purification and characterization of fusion proteins. (**A**) 12% SDS-PAGE confirming the purification of (4D5scFv)_2_-Bn (**a**, 71 kDa) and 425scFv-Bs (**a**', 40 kDa), Coomassie Brillian Blue R-25 stained gel; standard protein marker (M) and fusion protein lanes (P) are shown. (**B**) RNAse activity of (4D5scFv)_2_-Bn (solid line) compared with activity of free barnase (dashed line) and evaluated with acid-insoluble RNA precipitation assay. (**C**) Inhibition of free barnase by 425scFv-Bs (solid line) compared with inhibition by free barstar (dashed line).

Targeting fusion proteins were produced in *E. coli* and purified as described in Materials and Methods. The proteins obtained were of the expected molecular weight and homogeneity according to SDS-PAGE (*figure 3A*). The dissociation constant of the (4D5scFv)_2_-Bn from the purified p185^HER2-ECD^ was ∼2.2 nM. This value agrees well with that of the parental 4D5scFv antibody (∼5.2 nM). The enzymatic activity of the prepared (4D5scFv)_2_-Bn assessed by acid-insoluble RNA precipitation assay [16] was ∼8% of the native barnase activity (*figure 3B*). For comparison, the activity of barnase fused to different proteins has been reported to be ∼75% for each enzyme molecule in 4D5scFv-dibarnase protein [7] and ∼80% for barnase fused to exotoxin A from *Pseudomonas aeruginosa* [20]. Presumably, fusion of two antibody molecules to barnase results in steric hindrance effect and concomitant decrease of the RNAse activity.

The dissociation constant of the (4D5scFv)_2_-Bn from the purified EGFR was ∼2 µM. The pattern of barnase inhibition by 425scFv-Bs was similar to that by free barstar (*figure 3C*). Thus, the barstar moiety of 425scFv-Bs fusion protein retained its functionality.

### Visualizing fluorescent modules: conjugation of QDs with the BBS proteins

The following QD conjugates with the BBS proteins were obtained for subsequent use as visualizing fluorescent modules: QD_605_-Bs, QD_605_-Bn, QD_565_-Bs, and QD_565_-Bn. The QDs to be used in combination with barnase-containing targeting module ((4D5scFv)_2_-Bn) were conjugated with barstar, while those to be used with barstar-containing module (425scFv-Bs) were conjugated with barnase. Barnase and barstar were conjugated to QDs using EDC/NHS coupling chemistry. In the course of the reaction, the activated carboxyl groups on the surface of QDs react with ε-amino groups of the protein lysine residues as well as α-amino group of the N-terminal residue resulting in the formation of stable amide bonds.

**Figure 4:**
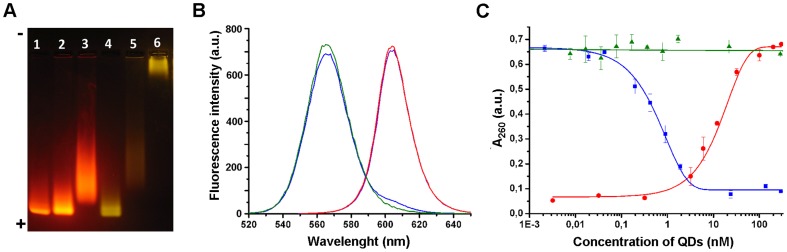
Characterization of QD-BBS protein conjugates. (**A**) Electrophoretic mobility of QDs and QD-conjugates in 1% agarose gel, in Tris-acetate-EDTA buffer (pH 7.4). QDs run from cathode (–) to anode (+). Lanes: 1 – QD_605_, 2 – mixture of QD_605_ and barstar (without EDC), 3 - QD_605_-Bs conjugate, 4- QD_565_, 5 –mixture of QD_565_ and barnase (without EDC), 6 - QD_565_-Bn conjugate. (**B**) Normalized fluorescence spectra of QD_565_ (blue line), QD_605_ (violet line) and their conjugates, QD_565_-Bn (green line) and QD_605_-Bs (red line). (**C**) The ribonuclease activity of QD_605_-Bn (red line and circles) and free barnase RNAse activity inhibition of QD_605_-Bs (blue line and square). QD_605_ (green line) do not affect ribonuclease activity of barnase.

The efficiency of the conjugation step was verified using agarose gel electrophoresis (*figure 4A*). Under the conditions exploited in the electrophoresis setup (pH 7.6), non-conjugated QDs are negatively charged, and thus migrate from cathode to anode (*figure 4A*, *lane 1, 4*). Upon addition of the BBS proteins to QD suspension, the electrophoretic mobility of the nanoparticles appears to be affected in different ways, depending on the protein added. The migration pattern of the mixture of QDs and negatively charged barstar (pI 4.6) is similar to that of free QDs (*figure 4A*, *lane 2*).In contrast, the addition of positively charged barnase (pI 9.8) that may bind to QDs due to electrostatic attraction resulted in a smeared migration pattern (*figure 4A*, lane 5).

Covalent conjugation of QDs with the BBS proteins using EDC cross-linker resulted in significant alteration of migration patterns. QD-Bs conjugates exhibited lower mobility than initial QDs or the mixture of QDs and free barstar, indicating successful functionalization of the QD surface (*figure 4A* *, lane 3*). QD-Bn conjugates barely leave the wells of the agarose gel, presumably due to neutralization of the negative charge of QD upon conjugation with barnase (*figure 4A* *, lane 6*).We also tested QDs and the BBS proteins for retention of their properties within the conjugates. Fluorescence spectroscopy measurements demonstrated that the emission spectra as well as quantum yields of QD fluorescence after conjugation to barnase or barstar remained virtually unaltered (*figure 4B*). The acid-insoluble RNA precipitation assay [16] indicates that the QD-Bn conjugates retain the functional properties of barnase, i.e., the ribonuclease activity, and QD-Bs conjugates retain barnase-binding capability and barnase inhibition activity (*figure 4C*).

### Live cell imaging

HER1- and HER2-overexpressing cancer cell lines, including A431 human epidermoid (3×10^6^ HER1 receptors/cell [21]) and human ovarian carcinoma SKOV-3 (2.6×10^5^ HER2/neu receptors/cell [22]) cells, were chosen to test specific staining of tumor cells with self-assembling fluorescent complexes. HER1- and HER2/neu-negative Chinese hamster ovarian cells (CHO) were used as controls.

**Figure 5:**
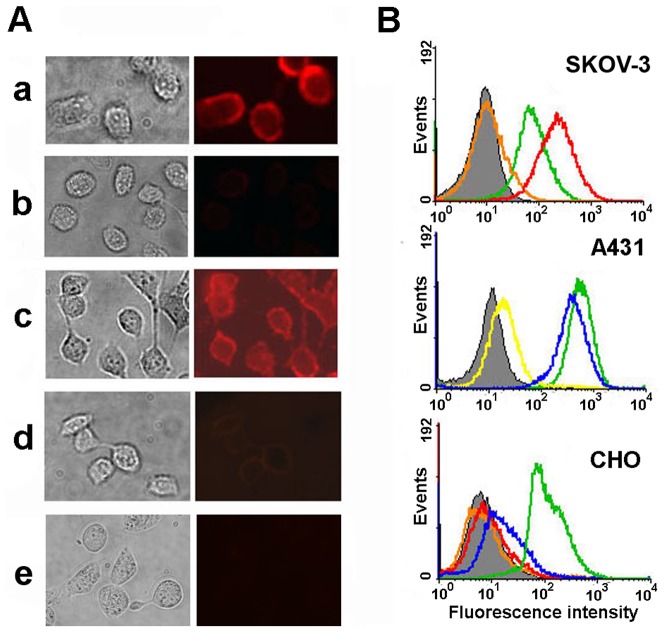
Live cell imaging. (**A**) Optical microscopy of HER1overexpressing A431 cells that were preincubated with QD_605_ (**a**), QD_605_-Bn (**b**), 425scFv-Bs and QD_605_-Bn (**c**). HER1-negative CHO cells were used as controls for staining with 425-Bs and QD_605_-Bn (**d**). As additional control competitive binding test of free 425scFv and anti-HER1 425scFv-Bs/QD_605_-Bn complex was carried out (**e**). **Left row**, bright-field image; **right row**, fluorescence image with 488 nm excitation and 605 nm emission peaks. (**B**) Flow cytometry of SKOV-3, A431 <$>\raster(70%)="rg1"<$> CHO cells incubated with QD_605_ (green line), QD_605_-Bn (yellow line), QD_605_-Bs (orange line), (4D5scFv)_2_-Bn and QD_605_-Bs (red line), 425scFv-Bsand QD_605_-Bn (blue line).

A major problem for cellular imaging with QDs is that QD probes tend to be ‘sticky’ and often bind non-specifically to cell membrane, proteins, and extracellular matrix [23], [24], [25], [26], [27], [28], [29]. The non-specific binding depends on surface properties of QD and the cell lines used. We evaluated the non-specific binding properties of the initial carboxylated QDs used for fluorescent probe construction. As shown in *figure 5* (*A-a and B – green line*), considerable non-specific binding is observed with QD_605_ incubated with SKOV-3, A431 and CHO cells at 4°C. We ascribe the level of non-specific binding to electrostatic interactions of the negatively charged QDs with the cell surface. Consequently, for directed and specific staining of tumor cells with QDs it is necessary not only to ensure proper targeting of the oncomarker of interest but also to decrease non-specific binding of QDs. To reduce non-specific binding, QDs are often modified with poly(ethylene glycol) (PEG), a hydrophilic polymer routinely used for such purposes in biological applications. But, in the present work the need for PEGylation can be avoided. Surprisingly, we found that QD conjugation to both the BBS proteins – barnase and barstar – decreases the non-specific binding of QDs to cell surface. When the cells were incubated with QD_605_-Bs or QD_605_-Bn at 4°C, weak or no fluorescent signal was detected on the cell surface, indicating that the QDs conjugated to BBS proteins have negligible non-specific binding (*figure 5* *, A-b and B-orange line for QD_605_-Bs or yellow line for QD_605_-Bn*). Similar results were obtained for QD_565_ and their conjugates. Thus, the use of the BBS proteins allowed not only binding of QDs with targeting antibodies but also reducing the non-specific binding of QDs to cell surface.

**Figure 6.Two:**
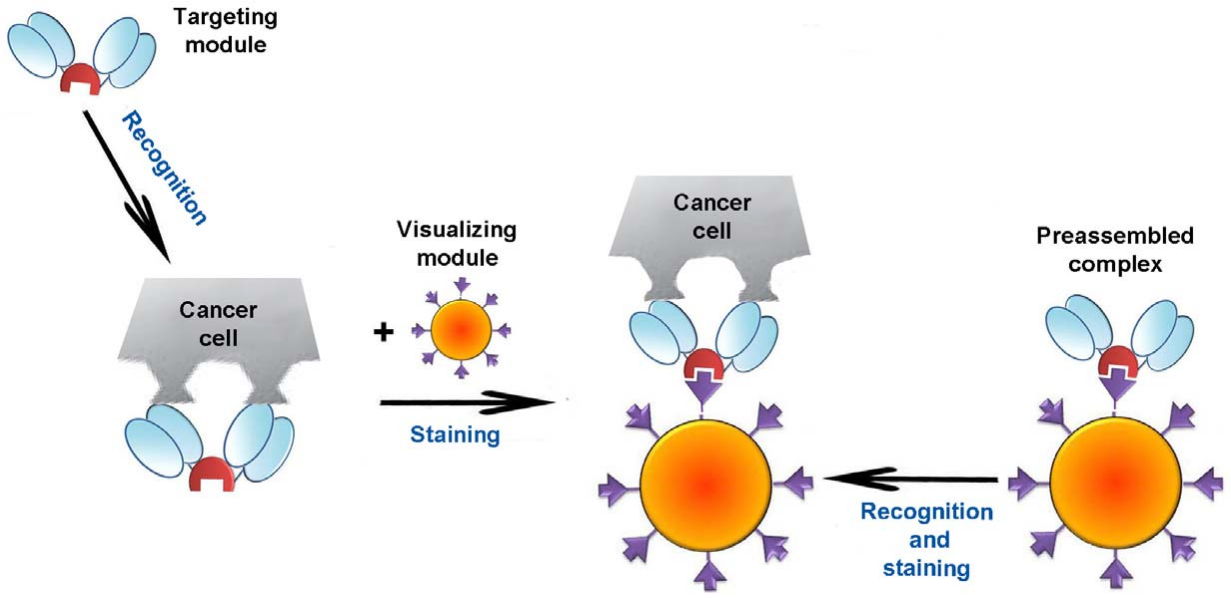
approaches for tumor cells imaging using QD-scFv antibody complexes based on barnase-barstar. Legends as in figure 1.

The ‘armament’of QD-BBS protein conjugates with targeting antibodies allowed specific labeling and imaging of tumor cells. The general strategy for specific tumor cell labeling by self-assembling fluorescent QD-scFv complex based on BBS system is illustrated in *figure 6* *(left)*. Tumor cells were pre-incubated with targeting fusion protein and then stained with QD-BBS protein conjugates. Visualizing module was connected to the cell-associated targeting module via the barnase-barstar interaction. Thus, the QD_605_-Bn conjugates effectively stained HER1 on the surface of A431 cells after the cells were incubated with the 425scFv-Bs targeting protein (*figure 5* *, A-c*). Likewise, SKOV-3 cells overexpressing HER2/neu were imaged by sequential treatment with (4D5scFv)_2_-Bn and QD_605_-Bs. Controls with HER1- and HER2/neu-negative CHO cells showed no staining, indicating the binding specificity of the targeted self-assembling complexes. In addition, when the A431 cells were treated with anti-HER1 fluorescent complex and free 425scFv (molar ratio 1∶2), the binding of complex to the cell membrane was completely blocked (*figure 5* *, A-e*). Analogous results were obtained using SKOV-3 cells, anti-HER2/neu complex and free 4D5scFv.

The use of QD_565_-Bs module makes it possible to perform cell imaging in the green spectral region. Thus, QD-scFv complexes with desired anti-tumor specificities and fluorescent spectra were obtained by combination of visualizing and targeting modules (Table 1).

In addition, tumor cells were successfully imaged by a one-step method using pre-assembled QD-scFv complexes. In this case, the targeting module (425scFv-Bs or (4D5scFv)_2_-Bn) and visualizing module (Bn-QD or Bs-QD, respectively) were pre-mixed for complex formation followed by incubation of the tumor cells with the resulting complexes. (*figure 6*).

**Figure 7:**
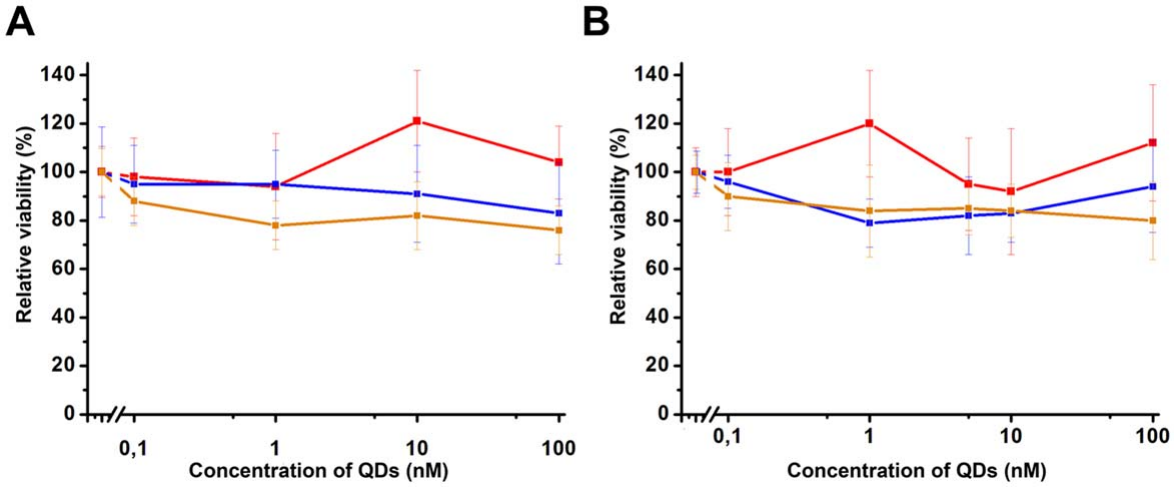
*In vitro* cytotoxicity analysis of used QD_565_ (A) and QD_605_ (B) probes. Relatively cell viability of SKOV-3 cells after treatment with initial QDs (red line), their conjugates with barstar (blue line) and their complex with 4D5scFv (orange line) are shown.

Although QDs are unique visualizing agent in terms of their photophysical and chemical properties, their deployment in biomedical applications are still hotly debated due to their potential cytotoxicity. We performed some preliminary experiments in order to estimate the cytotoxicity of the used quantum dotes and their conjugates and complexes. Cytotoxicity tests did not demonstrate any significant influence of QD_605_ and QD_565_ (as well as their derivatives) on the survival of SKOV-3 (*figure 7*). These results correspond well to the data published earlier demonstrating that quantum dots coated with polymer shell in concentrations required for visualization of particular surface receptors show no influence on the survival of cell cultures [30].

## Discussion

The exceptional physical and chemical properties of QDs enable multicolor and long-term imaging, thus substantially enhancing current methods of cancer cell fluorescent imaging and multiplex profiling of molecular tumor markers [2].

The HER1 and HER2/neu oncomarkers, the epidermal growth factor receptor family members, play a key role in the genesis and progression of certain types of tumors, including those of endometrium, ovary, breast, prostate and lung, and are clinically significant tumor markers [6].

For selective delivery to tumor cells overexpressing the biomarkers of interest, QDs need to be functionalized with targeting molecules. Monoclonal antibodies against HER1 [31] and HER2 [31], [32], [33], [34], [35] as well as EGF, the natural ligand of HER1 [36], [37], [38], have been successfully used as targeting moieties for the design of anti-tumor contrast agents based on QDs. However, full-length antibodies are relatively large, so the number of the antibodies that can be linked to the surface of QDs is limited and intra-tumoral distribution of the nanoparticles is impeded, which restricts the usage of full-length antibodies as QD targeting agents. The scFv appear to be more advantageous for generation of HER1- or HER2/neu-targeted QD probes, as they are smaller than full-length antibodies but retain antigen specificity and high binging affinity of parental antibodies. Other advantages of scFv antibodies as targeting modules include the relative simplicity of their production in bacteria, low immunogenicity, and lack of the antibody effector function [17].

Previous studies have revealed the feasibility of in vitro and in vivo imaging of tumor cells using nanoparticles conjugated with scFv antibodies targeting HER1 [38] or HER2/neu [39].

The use of self-assembling adaptors (small ‘sticky’ molecules that bind to each other with high efficiency and specificity but do not form homodimers) is a more promising approach to the QD-antibody binding than direct conjugation. The formation of complexes involving these molecules has no considerable effect on antibody affinity and allows for easy preparation of diverse combinations of antibodies with different specificities and QDs with various fluorescence spectra.

In this study, we have used the system based on a very specific and strong non-covalent (namely electrostatic) interaction between two proteins, barnase and barstar, as self-assembling molecular adaptors. The binding affinity of barnase and barstar is comparable to that of the (strept)avidin–biotin system, the strongest known among bio-molecules.

BBS was chosen because of several remarkable properties essential for the design of targeting modules. (**i**) Biotin is not a peptide, its attachment to proteins, e.g. antibodies, by gene engineering methods is not possible. Chemical linking is chaotic, usually nonspecific in terms of attachment geometry and often requires sophisticated post-modification separation of components. By contrast, both components of BBS are genetically encoded, which enables the creation of targeting modules as fusion proteins encoded by a single gene expressed in bacterial systems [15].Barnase and barstar are oppositely charged and thus allow us to choose the most suitable BBS protein for each antibody used for construction of targeting modules. That helps avoid complications associated with protein isolation, e.g., mutual ‘sticking’ of the domains of the obtained recombinant protein. In addition, barnase as a constituent of recombinant proteins can act as a molecular chaperone ensuring their correct folding [40]. (**ii**) Neither of the BBS proteins has analogues in mammals, which decreases the non-specific background when this system is used *in vivo*. On the contrary, the (strept)avidin-biotin system could not be properly used *in vivo* because biotin, also known as vitamin H, is widely present in the blood and tissues of mammals and may interfere with biotinylated agents during the drug's administration. (**iii**) The small size of both the BBS proteins suggests lower immunogenicity than that of larger proteins like streptavidin. In addition, the closest homologue of barnase, binase (84% homology), does not induce any T-cell immune response [41], so one would accordingly expect the same for barnase.

In this work, additional advantages of the BBS as a system for QD binding to targeting agents were determined: (i) binding of small proteins barnase and barstar to the surface of QDs significantly decreases non-specific interactions of QDs with the cell membrane; (ii) the BBS system allows one-step and two-step cell staining, in contrast to the (strept)avidin–biotin system, which is often inefficient when used in a one-step protocol [42]. According to previous studies [42], [43], in some cases the pre-assembled QD–antibody complexes based on the (strept)avidin–biotin system do not directly label tumor cells.

We have obtained a series of visualizing modules (on the basis of QDs and the BBS proteins) with different photochemical characteristics which could be combined with targeting modules by design (comprising of scFv antibodies and the BBS proteins). That represents the implementation of the idea of ‘molecular LEGO bricks’ developed in our previous studies (*figure 1B*). Genetically encoded recombinant antibodies as targeting modules can be characterized in detail and then combined with various QDs without loss of protein activity and specificity, which enables changing the target to be detected by using an appropriate targeting module.

The developed approach employing the BBS system for binding fluorescent nanoparticles to targeting anti-tumor scFv antibodies is universal and can be used for design of similar constructions containing QDs with other specificities and fluorescence spectra. In addition, the obtained conjugates of QDs and BBS proteins can be combined with molecules and nanoparticles of different nature [14].

In conclusion, we have obtained anti-HER1 and anti-HER2/neu QD-scFv complexes using the BBS system for binding QDs with targeting antibodies and studied the interactions of these complexes with cultured tumor cells. Our studies confirm that the fluorescent complexes bind efficiently to the target oncomarkers and exhibit low non-specific binding to cell membranes. Staining of the cells with the complexes can be carried out as a one-step procedure. The assembly of the complex realizes the principle of ‘molecular LEGO bricks’, and allows for combination of the targeting and visualization functions simply by varying the corresponding modules of the fluorescent complex.
